# Mechanical thrombectomy: can it be safely delivered out of hours in the UK?

**DOI:** 10.1186/s12883-020-01909-8

**Published:** 2020-09-01

**Authors:** Jake Weddell, Emma Parr, Stacey Knight, Girish Muddegowda, Indira Natarajan, Jayan Chembala, Phillip Ferdinand, Nasar Ahmad, Zoltan Pencz, Saad Rana, Anushka Warusevitane, Changez Jadun, Sanjeev Nayak, Zafar Hashim, Albin Augustine, Julius Sim, Christine Roffe

**Affiliations:** 1 Department of Stroke Medicine, Royal Stoke University Hospital, Newcastle Road, Stoke-on-Trent, ST4 6QG UK; 2 School of Medicine, Keele University, Newcastle-under-Lyme, ST5 5BG UK; 3 Department of Radiology, Royal Stoke University Hospital, Newcastle Road, Stoke-on-Trent, ST4 6QG UK; 4 Department of Anaesthetics, Royal Stoke University Hospital, Newcastle Road, Stoke-on-Trent, ST4 6QG UK

**Keywords:** Stroke, Mechanical thrombectomy, Large vessel occlusion

## Abstract

**Background:**

Mechanical thrombectomy was approved by NICE as a treatment for stroke in 2016. However, most of the evidence is from studies conducted during working hours. Only few centres in the UK perform thrombectomies out-of-hours. The Royal Stoke University Hospital (RSUH) has offered thrombectomies over 24 h (24/7) since 2010. The aim of this service review is to compare the outcomes for patients treated in regular working hours to those treated outside normal working hours within this unit.

**Methods:**

This retrospective service analysis includes all patients treated with mechanical thrombectomy at RSUH since the start of the service in January 2010 to June 2019. Data on key demographics, timings, procedural complications, and long-term outcomes including death and disability at 90 days were collected. In-hours was defined as the time between 8:00–17:00 h, Monday to Friday; out-of-hours was defined as any time outside this period.

**Results:**

In total, 516 mechanical thrombectomies were performed in this time period; data were available on 501 of these. Successful recanalization (TICI 2b/3) was achieved in 86% of patients. By 90 days 96 (19%) had died and 234 (47%) were functionally independent (modified Rankin Scale score ≤ 2). 211 (42%) of the procedures were performed in-hours and 290 (58%) out-of-hours. Door-to-CT and door-to-groin times were significantly longer out-of-hours than in-hours, but thrombectomy duration was significantly shorter. There were no significant differences in complications and short- and long-term outcomes.

**Conclusion:**

Mechanical thrombectomy was delivered safely and effectively 24/7 in this UK hospital, with no difference in clinical outcomes.

## Background

The development of mechanical thrombectomy has revolutionized the treatment of large-vessel occlusive stroke. In patients presenting up to 6 h from stroke onset, mechanical thrombectomy improves the absolute rate of functional independence by approximately 20%, compared to best medical therapy [1]. It has been approved by the National Institute for Health and Care Excellence (NICE) in 2016 [2]. Recent trials have demonstrated that perfusion imaging can increase the time window for mechanical thrombectomy to up to 24 h in selected patients [3, 4]. It is estimated that around 10% of stroke patients may benefit from mechanical thrombectomy, representing approximately 10,000 patients a year in the UK alone [5].

Delivering mechanical thrombectomy services to eligible patient populations has proven challenging. In the UK, a scarcity of experienced operators, lack of existing infrastructure and geographical limitations have severely hampered service delivery [6]. As such, mechanical thrombectomy is only provided at a limited number of specialist neuroscience centres and mostly within normal working hours or on an ad hoc basis. The paucity of 24-h (24/7) UK thrombectomy centres has contributed to the UK falling behind internationally in the provision of mechanical thrombectomy. In a 6-month period in 2018, just 478 mechanical thrombectomies were performed in the UK, compared to over 9000 in Germany and over 4500 in France [7].

The Royal Stoke University Hospital (RSUH) was the first UK centre to perform mechanical thrombectomy for ischaemic stroke on a regular basis and the first to deliver this 24/7. A review of the service performed in 2014 demonstrated endovascular therapy-based services could be safely and effectively delivered in a UK setting, with nearly 50% of patients having a favorable outcome [8]. Thrombectomy was conducted 24/7 from the outset, with ad hoc availability of staff in the first few years, and a formal rota since 2016. While all staff are on site in-hours, out-of-hours the stroke physician, the radiographer, the theatre nurse, and the neurointerventionist have to travel in. Anaesthesia is covered by a consultant neuroanaesthetist in the day, and the registrar on call with consultant back-up out-of-hours. The aim of this study was to establish whether, in the context of the current service offered, there are differences in thrombectomy outcomes for patients admitted to RSUH within normal working hours compared to with those admitted outside normal working hours.

## Methods

A service review was undertaken of all patients who were treated with mechanical thrombectomy for large-vessel occlusive stroke between the start of January 2010 and the end of June 2019 at RSUH, a large comprehensive stroke centre in the West Midlands (UK) admitting about 1200 stroke patients per year from the local population and as transfers for neurointervention. Data were extracted from a prospective database of all patients who receive mechanical thrombectomy. In total 516 mechanical thrombectomy procedures were performed in this time period; key data were missing in 15 of these patients, leaving 501 included in the complete analysis. Data extracted included: age, sex, stroke severity assessed by the National Institutes for Health Stroke Scale (NIHSS) at onset, risk factors for stroke, use of thrombolytic agents, anaesthetic strategy, affected vessel, timings, post-procedural complications, and disability, assessed by the modified Rankin Scale (mRS) at 90 days. For patients who were intubated or in coma, a NIHSS of 35 was assigned. Death was recorded as a NIHSS of 42 (the highest numerical score on the scale) and an mRS score of 6.

Background characteristics and the NIHSS were assessed on admission by either a consultant stroke physician or a stroke nurse. All formal imaging was reported by a consultant neuroradiologist. The mRS score at 90 days and post-procedure complications were assessed over the phone or in person by a consultant stroke physician or an advanced nurse practitioner. Occlusion location was defined as most proximal occlusion present on digital subtraction angiography. Symptomatic intracranial haemorrhage was defined using Safe Implementation of Thrombolysis in Stroke Monitoring Study (SITSMOST) criteria as a local or remote parenchymal haemorrhage type 2 on the 22–36 h post-treatment head scan, combined with a neurological deterioration of four or more points from the lowest NIHSS within 24 h or leading to death [9]. Malignant cerebral oedema was defined as cerebral oedema causing midline shift with associated clinical deterioration of 4 or more NIHSS points. Groin haematoma was defined as a haematoma requiring medical intervention, including blood transfusion or fibrin injection.

In-hours admission was defined as between 8:00–17:00 from Monday to Friday, excluding public holidays. This was chosen as the time period where the full stroke, interventional and neuroanaesthetic teams are present on the hospital site. Patients admitted outside this time window were classified as an out-of-hours admission. For those patients who suffered a stroke whilst an inpatient at RSUH, onset time was used to determine whether the stroke was in-hours or out-of-hours.

### Statistics

Demographic and clinical characteristics of patients were summarized through frequencies and percentages or means and standard deviations. As this is a service review, for the purpose of statistical testing the population was defined as past and future stroke patients admitted to RSUH, rather than the broader population of stroke patients in the UK. The assumptions of all statistical analyses were checked. Baseline comparisons were made through unrelated *t* tests for numerical variables and through Pearson chi-square tests for binary variables. Comparisons of procedural factors and short- and long-term outcomes were made between in-hours and out-of-hours patients in a multivariable statistical model, controlling for baseline demographic and clinical characteristics (see Table 1). Numerical outcomes were analysed through analysis of covariance, with differences expressed as mean differences, and binary outcomes through multivariable logistic regression, with differences expressed as odds ratios (ORs). The OR indicates the increase or decrease in the odds of an outcome for a one-unit increase in the predictor variable (e.g. moving from female to male, or an additional year of age). In this instance, an OR greater than 1 indicates that the outcome was more likely among in-hours patients than among out-of-hours patients, and an OR less than 1 that it was less likely. Statistical significance was set at *p* ≤ 0.05 (two-tailed) and 95% confidence intervals (CIs) were calculated for between group estimates. No correction was made for multiple testing.

**Table 1:** Baseline demographic and clinical details

	Total (***n*** = 501)	In-hours (***n*** = 211)	Out-of-hours (***n*** = 290)	***p*** value*
Age: mean (SD)	66.7 (13.7)	68.6 (12.6)	65.3 (14.2)	0.008
Sex: n (%) males	262 (52.3)	100 (47.4)	162 (55.9)	0.061
Hypertension: n (%)	252 (50.3)	93 (54.8)	159 (44.1)	0.017
Atrial fibrillation: n (%)	140 (27.9)	56 (26.5)	84 (29.0)	0.550
Hyperlipidaemia: n (%)	134 (26.7)	56 (26.5)	78 (26.9)	0.929
Diabetes: n (%)	80 (16.0)	29 (13.7)	51 (17.6)	0.246
Previous stroke/TIA: n (%)	76 (15.2)	31 (14.7)	45 (15.5)	0.799
Coma pre procedure: n (%)	17 (3.4)	7 (3.3)	10 (3.4)	0.936
Thrombolysis: n (%)	368 (73.5)	155 (73.5)	213 (73.4)	0.998
NIHSS at onset: median; mean (SD)	18.0; 18.3 (7.0)	18.0; 18.1 (7.1)	18.0; 18.4 (6.9)	0.718
Anterior circulation lesion: n (%)	447 (89.2)	189 (89.6)	258 (89.0)	0.828**
CCA: n (%)	6 (1.2)	4 (1.9)	2 (0.7)	
ICA: n (%)	158 (31.5)	64 (30.3)	94 (32.4)	
ACA: n (%)	1 (0.2)	1 (0.5)	0 (0.0)	
M1: n (%)	239 (47.7)	98 (46.4)	141 (48.6)	
M2: n (%)	37 (7.4)	19 (9.0)	18 (6.2)	
M3: n (%)	6 (1.2)	3 (1.4)	3 (1.0)	
Posterior circulation lesion: n (%)	54 (10.8)	22 (10.4)	32 (11.0)	
Vertebral artery: n (%)	4 (0.8)	2 (0.9)	2 (0.7)	
Basilar artery: n (%)	47 (9.4)	18 (8.5)	29 (10.0)	
PCA: n (%)	3 (0.6)	2 (0.9)	1 (0.3)	

*ACA* anterior cerebral artery, *CCA* common carotid artery, *ICA* internal carotid artery, *TIA* transient ischaemic attack, *M1, M2, M3* middle cerebral artery segments 1, 2 and 3, *NIHSS* National Institutes for Health Stroke Scale, *PCA* posterior cerebral artery, *SD* standard deviation

* *p* values are derived from an unrelated *t* test for continuous variables and a Pearson chi-square test for nominal variables

** *p* value for a comparison across groups of anterior versus posterior circulation lesions

In addition to the main comparison between in-hours and out-of-hours, two exploratory analyses were conducted: between day (08:00–21:59) and night (22:00–07:59) and between weekday and weekend. These comparisons are presented with 95% CIs, but hypothesis tests were not conducted in view of the exploratory nature of these analyses (see supplementary tables).

As this was a service review, patients and the public were not involved in the design, conduct, reporting, or dissemination plans.

## Results

### Demographics

**Fig. 1:**
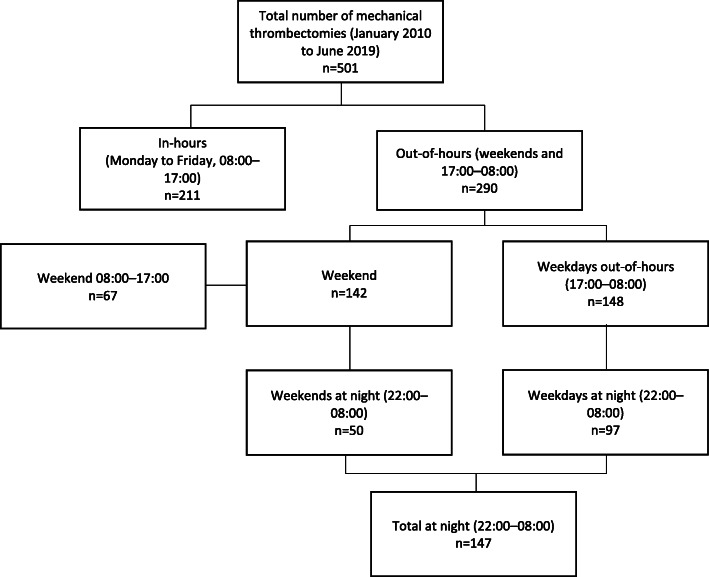
Flowchart showing number of patients treated in-hours vs out of hours, day vs night and weekend vs weekday

In total, 501 patients treated with mechanical thrombectomy between 2010 and 2019 were analysed (Table 1). Of these patients, the majority were treated outside of normal working hours, with 290 (58%) treated in this time period and 211 (42%) treated in-hours (detailed breakdown in Fig. 1). The mean age of included patients was 66.7 (standard deviation [SD] 13.7), and 262 (52%) were male. Most strokes were severe, with a mean (SD) NIHSS score of 18.3 (7.0) and median of 18.0. The majority (89%) affected the anterior circulation. Patients admitted out-of-hours were significantly younger and more likely to have a prior diagnosis of hypertension than those admitted in-hours. There were no other significant differences in demographic or clinical characteristics between the two groups.

### Timings

The mean time from hospital admission (door) to the end of the thrombectomy was 205 min with a mean 34 min time lapse between door and computed tomography (CT) of the head, a mean of 113 min between CT and start of the procedure (groin time defined as femoral artery puncture) and a mean procedure duration of 64 min (Table 2). Patients admitted during working hours had a significantly lower door-to-CT time, resulting in a corresponding lower door-to-groin time. Patients presenting out-of-hours had a significantly reduced procedure time, but there was no significant difference in door-to-procedure-end-times between the two groups.

**Table 2:** Timelines from onset to completion of the procedure

	Total (***n*** = 501)	In-hours (***n*** = 211)*	Out-of-hours (***n*** = 290)*	Adjusted mean difference (95% CI); ***p*** value**
Onset to door: median; mean (SD)	108.0; 172.1 (195.1)	109.0; 169.8 (192.4)	107.0; 173.9 (197.5)	−2.6 (−37.2, 32.1); .884
Door to CT***: median; mean (SD)	23.0; 34.1 (45.4)	21.0; 26.3 (22.7)	24.0; 40.0 (56.4)	14.9 (6.7, 23.2); <.001
CT to groin***: median; mean (SD)	104.5; 113.0 (63.6)	89.5; 108.6 (71.8)	111.0; 116.3 (56.6)	8.7 (−3.0, 20.4); .143
Procedure time: median; mean (SD)	55.0; 63.8 (42.5)	60.0; 69.5 (44.0)	53.0; 59.6 (40.9)	−10.8 (− 18.5, −3.2); .006
Door to groin: median; mean (SD)	124.0; 138.2 (85.5)	110.0; 124.9 (73.7)	135.5; 148.0 (92.1)	25.6 (11.3, 39.9); <.001
Door to procedure end: median; mean (SD)	184.0; 204.9 (105.4)	181.0; 196.5 (93.0)	188.5; 211.1 (113.4)	16.4 (−1.7, 34.4); .075

*CI* confidence interval, *SD* standard deviation

End of procedure is defined as the time of the final intracranial angiogram

* Denominators differ between variables owing to missing or not applicable values

** Out-of-hours minus in-hours; a positive difference indicates time was greater for out-of-hours. Adjusted for age, sex, hypertension, atrial fibrillation, hyperlipidaemia, diabetes mellitus, previous stroke/TIA, coma, NIHSS at onset, anterior/posterior circulation lesion

*** In total 40 patients were transferred straight to the neurointervention suite and received no imaging at RSUH prior to thrombectomy

Table S2 shows the estimates in respect of the exploratory comparisons between day and night and weekday and weekend.

### Procedural factors

Details of the procedure are shown in Table 3. Advanced imaging (CT perfusion) was used in 11% of cases. The majority of cases (74%) were conducted under general anaesthesia. Recanalization was successful (thrombolysis in cerebral infarction (TICI) score of 2b or 3) in 86% of cases, and was achieved at first pass in 45% of cases. CT perfusion (OR = 2.49) and successful recanalization (OR = 1.93) were more likely in-hours than out-of-hours, but first-pass recanalization (OR = 0.64) was less likely. Other differences were non-significant.

**Table 3:** Details of the procedure

	Total (***n*** = 501)	In-hours (***n*** = 211)	Out-of-hours (***n*** = 290)	Adjusted odds ratio (95% CI); ***p*** value*
CT perfusion: n (%)	56 (11.2)	35 (16.6)	21 (7.2)	2.49 (1.36, 4.54); .003
General anaesthetic: n (%)	371 (74.1)	158 (77.1)	213 (77.2)	1.08 (0.69, 1.70); .744
Successful recanalization: n (%)	431 (86.0)	188 (90.4)	243 (85.3)	1.93 (1.07, 3.50); .030
First pass recanalization: n (%)	223 (44.5)	83 (42.1)	140 (53.2)	0.64 (0.43, 0.94); .023
Intracranial vasospasm: n (%)	7 (1.4)	3 (1.4)	4 (1.4)	1.37 (0.28, 6.85); .699
Dissection: n (%)	33 (6.6)	18 (8.6)	15 (5.3)	1.92 (0.91, 4.03); 086

Percentages are valid percentages (based on non-missing values) Successful recanalization was defined as a thrombolysis in cerebral infarction (TICI) score of 2b or 3. An odds ratio greater than 1 indicates that the outcome was more likely among in-hours patients than among out-of-hours patients, and an odds ratio less than 1 that it was less likely

* Out-of-hours as reference category. Adjusted for age, sex, hypertension, atrial fibrillation, hyperlipidaemia, diabetes mellitus, previous stroke/TIA, coma, NIHSS at onset, anterior/posterior circulation lesion

Table S3 shows the estimates in respect of the exploratory comparisons between day and night and weekday and weekend. Differences in in-hospital timelines somewhat more pronounced with longer delays for all steps at night compared with the primary analysis (in hours vs out of hours), while differences were less marked in the weekday vs weekend comparison.

### Long- and short-term outcomes

Outcomes and complications up to 90 days are shown in Table 4. Eight patients (2%) had a symptomatic intracerebral haemorrhage (SITS criteria). At 90 days 234 (47%) were independent in activities of daily living (mRS ≤ 2). Short- and long-term outcomes did not differ significantly between the two groups, though the low number of events in some of the binary variables means that the statistical comparisons would have low power and attention should be focused on the magnitude of the differences.

**Table 4:** Complications and outcomes up to 90 days

	Total (***n*** = 501)	In-hours (***n*** = 211)	Out-of-hours (***n*** = 290)	Adjusted difference (95% CI); ***p*** value*
NIHSS at 1 week: median; mean (SD)	7.0; 13.0 (14.0)	6.0; 12.1 (13.0)	7.0; 13.7 (14.6)	1.2 (− 1.2, 3.5); .334
Renal failure; n (%)	8 (1.6)	4 (1.9)	4 (1.4)	1.06 (0.22, 4.98); .946
Groin haematoma: n (%)	8 (1.6)	5 (2.4)	3 (1.0)	1.93 (0.43, 8.65); .392
Malignant middle cerebral artery syndrome: n (%)	44 (8.8)	12 (5.7)	32 (11.1)	0.52 (0.24, 1.14); .102
Hemicraniectomy: n (%)	13 (2.6)	5 (2.4)	8 (2.8)	1.42 (0.37, 5.44); .606
Symptomatic intracerebral haemorrhage: n (%)	8 (1.6)	3 (1.4)	5 (1.7)	0.85 (0.19, 3.73); .827
Subarachnoid haemorrhage: n (%)	45 (9.0)	22 (10.9)	23 (8.2)	1.28 (0.68, 2.41); .449
Deep vein thrombosis within 90 days: n (%)	12 (2.4)	7 (3.3)	5 (1.7)	1.95 (0.57, 6.64); .287
Pulmonary embolism within 90 days: n (%)	15 (3.0)	7 (3.3)	8 (2.8)	1.45 (0.48, 4.34); .509
Stroke within 90 days: n (%)	10 (2.0)	2 (1.0)	8 (2.8)	0.36 (0.07, 1.78); .210
Death at 90 days: n (%)	96 (19.2)	34 (16.1)	62 (21.4)	0.61 (0.36, 1.02); .061
Functional independence at 90 days: n (%)	234 (46.7)	100 (47.4)	134 (46.2)	1.07 (0.72, 1.58); .746

Percentages are valid percentages (based on non-missing values). Functional independence is defined as modified Rankin Scale score of 0–2. Symptomatic intracerebral haemorrhage is defined as per the SITS-MOST criteria

* Difference expressed as odds ratio, with out-of-hours as reference category, except for NIHSS, where it is mean difference (out-of-hours minus in-hours). Adjusted for age, sex, hypertension, atrial fibrillation, hyperlipidaemia, diabetes mellitus, previous stroke/TIA, coma, NIHSS at onset, anterior/posterior circulation lesion

With respect specifically to mortality at 90 days, there was no significant difference between in-hours and out-of-hours (Table 4). The significant predictors of mortality within this analysis were age (OR = 1.04), NIHSS at onset (OR = 1.12), diabetes (OR = 2.60), thrombolysis (OR = 0.53), posterior circulation lesion (OR = 2.51); *p* ≤ .024 in each case. The goodness of fit for the model (Nagelkerke pseudo-*R*^2^) was .234.

Table S4 shows the estimates in respect of the exploratory comparisons between day and night and weekday and weekend.

### Year by year

Comparison of cases performed, timings and outcomes on a year-by-year basis are shown in Table 5. Service demand at RSUH has increased significantly over the 10-year period analysed, from 18 cases in 2010 to 86 in 2018. Mean door-to-groin times show a trend of improvement from 190 min in 2010 to 127 min in 2019. Trends in relation to death at 90 days and functional independence at 90 days are less clear.

**Table 5:** Year-by-year comparison

Year	Cases (***n*** = 501)	Death at 90 days (***n*** = 96); n (%)	Functional independence at 90 days (***n*** = 234); n (%)	Door to groin timings; median; mean (SD)
2010	18	2 (11.1)	9 (50.0)	162.5; 190.3 (112.7)
2011	27	2 (7.4)	17 (63.0)	135.0; 141.7 (55.6)
2012	40	7 (17.5)	15 (37.5)	142.5; 143.9 (74.8)
2013	53	7 (13.2)	24 (45.3)	127.0; 154.9 (113.8)
2014	44	12 (27.3)	17 (38.6)	136.0; 149.0 (74.3)
2015	58	14 (24.1)	31 (53.4)	122.5; 137.9 (84.4)
2016	68	15 (22.1)	35 (51.5)	114.0; 126.6 (81.9)
2017	60	12 (20.0)	31 (51.7)	108.0; 131.2 (107.3)
2018	86	15 (17.4)	39 (45.3)	118.0; 128.5 (72.3)
2019 (to June)	47	10 (21.3)	16 (34.0)	118.0; 127.0 (58.3)

*SD* standard deviation

### Comparison to the Swift prime trial

A cohort of patient from our database who met the SWIFT PRIME trial entry requirements were identified (Table 6). Patients eligible for SWIFT PRIME in our dataset had a similar mortality (11% vs 9%) and functional independence (59% vs 60%) rate to those treated in the original trial [10].

**Table 6:** Swift prime trial comparison

	Royal Stoke University Hospital (***n*** = 501)	Swift Prime trial
Total eligible	142	98
Mortality at 90 days	15 (11%)	9 (9%)
Functional independence at 90 days	85 (59%)	59 (60%)

Number of patients eligible for treatment if the Swift-Prime trial inclusion criteria had been applied and outcomes at 90 days. Eligibility of 142 patients was based upon: age > 18 and < 80 (*n* = 423); NIHSS > 8 and < 30 (*n* = 422); intravenous tissue plasminogen activator given (*n* = 368); internal carotid artery or middle cerebral artery segment 1 occlusion (*n* = 397); onset to procedure end < 6 h (*n* = 294)

## Discussion

In this service review of over 500 patients treated with mechanical thrombectomy we have demonstrated that a 24/7 mechanical thrombectomy service can be delivered safely and effectively in this comprehensive stroke centre in a UK NHS hospital, with no significant difference in short- or long-term outcomes for patients admitted in-hours versus out-of-hours. This includes rates of functional independence and death at 90 days, early post-procedural complications, and symptomatic intracerebral haemorrhage. However, patients admitted out-of-hours had significantly longer door-to-groin times, with delays at all points of the treatment pathway contributing to this effect. Procedure times were, however, shorter for out-of-hours patients.

A previous service review conducted at RSUH of 106 stroke patients treated between 2009 and 2013 demonstrated that endovascular therapy could be delivered safely and effectively within this UK setting [8]. Since this was published many changes have occurred in mechanical thrombectomy, with the publication of the landmark positive randomized controlled trials in 2015 [11] and the use of CT perfusion software in assessing the extent of the viable penumbra in late presenters [3, 4]. This has greatly expanded the demand for mechanical thrombectomy at our unit, with 86 mechanical thrombectomies performed in 2018 compared to 18 in 2010.

In trials establishing mechanical thrombectomy as a viable treatment for acute ischaemic stroke, there was little impact on rate of death in comparison to best medical therapy [11]. Our study reported an overall mortality of 19% at 90 days, which is higher than the 15% reported in our previous service review [8], with similar rates of functional independence at 90 days: 48% then versus 47% now. The difference in mortality is likely due to changes in patient selection (mean age 64 years then and 66.8 years now), with increasing age being significantly associated with mortality at 90 days in our multiple logistic regression analysis.

There are no published national data for 90-day outcomes after thrombectomy, making it difficult to gauge how our hospital’s performance compares with others in the UK. A recent service review from the Walton Centre in Liverpool showed a 33% mortality after thrombectomy; however, this review only analysed 48 patients in total [12]. Data from the mechanical thrombectomy service at the Royal Victoria Hospital Belfast showed an overall mortality rate of 22%, of 214 patients treated between 2014 and 2017 [13]. A meta-analysis of 8 thrombectomy RCTs in 2015 showed a mortality of 16% [14], and in the more recent 5 large RCTs using modern devices mortality ranged from 9 to 21% [1]. Mortality in the 33 patients in the thrombectomy group in the UK PISTE RCT was 21%. Only 147 patients treated at our centre over the 10-year time period would be eligible for thrombectomy under SWIFT PRIME criteria. Those who did undergo thrombectomy had a similar mortality rate to those in the original trial (11% vs 9%) [10]. Because of these more stringent inclusion criteria, mortality in RCTs tends to be lower than in unselected clinical cases.

A recent whole-country review of thrombectomy services in Germany between 2015 and 2018 found a mortality rate of 29% and a functional independence rate of 37% at 90 days [15]. These findings are most likely a result of inclusion of high-risk patients – such as those with basilar artery occlusions, significant co-morbidities, or higher initial mRS scores –combined with an increase in treatment of patients with more borderline inclusion criteria.

Ischaemic stroke occurrence shows a diurnal variation, with the majority of strokes occurring in the morning between 9:00 am and noon [16]. However, a recent study from the USA showed that the most common time for mechanical thrombectomy to be performed was in the evening between 21:00 and 22:00, with 58.7% of mechanical thrombectomies performed outside of normal working hours [17]. This was similar to our data, where the majority of mechanical thrombectomies were performed out-of-hours. Currently in the UK, few centres offer a 24/7 service for mechanical thrombectomy, potentially excluding a large number of eligible patients who may benefit from this procedure.

Despite the possibility of treating patients up to 24 h post stroke onset with CT perfusion, the adage ‘time is brain’ still remains true. This makes minimizing door-to-groin times essential for improving long-term functional outcomes. Additionally, this metric acts as a surrogate marker of effective service delivery. In our previous service review only door-to-procedure-end time was recorded; on this composite metric we have significantly improved with a median reduction of over 60 min. Our median door-to-groin times are, at 124 min, better than the UK national average (149 min) in the 2018/19 data cut of the UK National Sentinel Stroke Audit [18], but considerably slower than the 64 min in a national audit of stroke care in the Netherlands [19]. Long door-to-groin times out-of-hours have been reported at other centres. Two recent studies of in-hours and out-of-hours thrombectomy from the United States [20] and Germany [21] reported similar delays in door-to-groin times (21 min and 20 min respectively), but also found worse early [21] and late [20] outcomes in patients treated out-of-hours. In contrast, another study from Germany, with no delay in door-to-groin time out-of-hours, found no difference in early or late outcomes following mechanical thrombectomy [22]. Outcomes were no different in- and out-of-hours in our hospital. This may be because delays in the early part of the pathway (door-to-groin) were compensated by more rapid procedure times at night.

On reviewing the exploratory analyses, differences in pathway timings were identified on comparison between day versus night; and weekend versus weekday, suggesting pathway delays were not limited to a specific time period. These pathway delays did not lead to major differences in long or short term outcomes.

This is the largest report of thrombectomy outcomes from a single centre in the UK, with a well-established service dating back to 2010 [8]. Our database demonstrated a high rate of follow-up and differences in baseline demographic and clinical characteristics were controlled for in the statistical analysis, thereby removing potential biasing of the in-hours versus out-of-hours comparisons. Limitations are that there were changes in the thrombectomy pathway in our hospital and in eligibility criteria for thrombectomy over the time period, with the development of a hub and spoke model with more remote transfers over time, and more extensive use of advanced imaging and inclusion of patients up to 24 h after onset during the final 2 years.

## Conclusion

Demand for mechanical thrombectomy procedures has increased dramatically over the past few years. Expanding evidence from randomized controlled trials has greatly contributed to this increase, and as the evidence continues to evolve, further increases in demand are expected. We have presented a service analysis of the longest running 24/7 mechanical thrombectomy service in the UK and analysed the effect of admission time on overall outcomes. The results of our study show that thrombectomy can be provided safely and effectively out-of-hours in our hospital, when fewer staff are on site. Further improvements could be made in our centre by speeding up the CT-to-groin time. This may require a service redesign.

Rolling out thrombectomy for extended hours, and eventually 24/7 nationally, will be a key step in addressing this shortfall in service provision in the UK.

## Supplementary information

**Additional file 1: Table S1.** Baseline demographic and clinical details, day versus night and weekday versus weekend. **Table S2.** Timelines from onset to completion of the procedure, day versus night and weekday versus weekend. **Table S3.** Details of the procedure, day versus night and weekday versus weekend. **Table S4.** Complications and outcomes up to 90 days, day versus night and weekday versus weekend.

## Data Availability

The datasets generated and analysed during the current study are not publicly available, but anonymised data may be available from the corresponding author on reasonable request.

## Abbreviations

ACA: Anterior cerebral artery
CCA: Common carotid artery
CI: Confidence interval
CT: Computed tomography
ICA: Internal carotid artery
mRS: Modified Rankin Scale
M1, M2, M3: Middle cerebral artery segments 1, 2 and 3
NICE: National Institute for Health and Clinical Excellence
NIHSS: National Institutes of Health Stroke Scale
OR: Odds ratio
PCA: Posterior cerebral artery
SD: Standard deviation
TIA: Transient ischaemic attack
TICI: Thrombolysis in cerebral infarct
