# Supplementary material for: Mechanical thrombectomy: can it be safely delivered out of hours in the UK?
Source: BMC Neurol. 2020 Sep 1;20:326. doi: 10.1186/s12883-020-01909-8 (PMC7461259; doi:10.1186/s12883-020-01909-8)
Supplement: Supplementary file 1 — Additional file 1: Table S1. Baseline demographic and clinical details, day versus night and weekday versus weekend. Table S2. Timelines from onset to completion of the procedure, day versus night and weekday versus weekend. Table S3. Details of the procedure, day versus night and weekday versus weekend. Table S4. Complications and outcomes up to 90 days, day versus night and weekday versus weekend. [file 12883_2020_1909_MOESM1_ESM.docx]

Supplementary tables

# Table S1 Baseline demographic and clinical details, day versus night and weekday versus weekend

|  | **Day vs night** | | **Weekday vs weekend** | |
| --- | --- | --- | --- | --- |
|  | **Day (n=354)** | **Night (n=147)** | **Weekday (n=359)** | **Weekend (n=142)** |
| Age; mean (SD) | 67.1 (13.7) | 65.7 (13.5) | 66.9 (13.7) | 66.1 (13.5) |
| Sex; n (%) males | 182 (51) | 80 (54) | 179 (50) | 83 (59) |
| Hypertension; n (%) | 164 (46) | 88 (60) | 178 (50) | 74 (52) |
| Atrial fibrillation; n (%) | 96 (27) | 44 (30) | 107 (30) | 33 (23) |
| Hyperlipidaemia; n (%) | 94 (27) | 40 (27) | 95 (27) | 39 (28) |
| Diabetes; n (%) | 52 (15) | 28 (19) | 56 (16) | 24 (17) |
| Previous stroke/TIA; n (%) | 52 (15) | 24 (16) | 57 (16) | 19 (13) |
| Coma pre-procedure; n (%) | 10 (3) | 7 (5) | 14 (4) | 3 (2) |
| Thrombolysis; n (%) | 266 (75) | 102 (69) | 265 (74) | 103 (73) |
| NIHSS at onset; mean (SD)* | 18.4 (7.0) | 18.0 (7.1) | 18.2 (7.2) | 18.5 (6.7) |
| Anterior circulation; n (%) | 322 (91) | 125 (85) | 318 (89) | 129 (91) |
| CCA; n (%) | 4 (1) | 2 (1) | 6 (2) | 0 (0) |
| ICA; n (%) | 104 (29) | 54 (37) | 112 (31) | 46 (32) |
| ACA; n (%) | 1 (<1) | 0 (<1) | 1 (<1) | 0 (0) |
| M1; n (%) | 176 (50) | 63 (43) | 166 (46) | 73 (51) |
| M2; n (%) | 33 (9) | 4 (3) | 28 (8) | 9 (6) |
| M3; n (%) | 4 (1) | 2 (1) | 5 (1) | 1 (1) |
| Posterior circulation; n (%) | 32 (9) | 22 (15) | 41 (11) | 13 (9) |
| Vertebral artery; n (%) | 4 (1) | 0 (0) | 4 (1) | 0 (0) |
| Basilar artery; n (%) | 25 (7) | 22 (15) | 35 (10) | 12 (9) |
| PCA; n (%) | 3 (1) | 0 (0) | 2 (1) | 1 (1) |

ACA = anterior cerebral artery; CCA = common carotid artery; ICA = internal carotid artery; TIA = transient ischaemic attack; M1, M2, M3 = middle cerebral artery segments 1, 2 and 3; NIHSS = National Institutes for Health Stroke Scale; PCA = posterior cerebral artery; SD = standard deviation

* 3 missing values

# Table S2 Timelines from onset to completion of the procedure, day versus night and weekday versus weekend

|  | **Day versus night** | | | **Weekday versus weekend** | | |
| --- | --- | --- | --- | --- | --- | --- |
|  | **Day (n=354)** | **Night (n=147)** | **Adjusted mean difference [night – day] (95% CI)** | **Weekday (n=359)** | **Weekend (n=142)** | **Adjusted mean difference [weekend – weekday] (95% CI)** |
| Onset to door  Median; mean (SD) | 120.0; 178.1 (210.2) | 101.0; 162.2 (150.3) | –27.0 (–64.7, 10.8) | 116.0; 169.2 (175.2) | 106.0; 185.1 (239.4) | 16.3 (–21.3, 53.9) |
| Door to CT*  Median; mean (SD) | 21.0; 28.6 (28.5) | 27.0; 47.2 (69.8) | 16.9 (8.0, 25.8) | 22.0; 33.0 (48.8) | 24.0; 36.6 (41.8) | 4.9 (–4.2, 14.0) |
| CT to groin*  Median; mean (SD) | 96.0; 102.4 (68.9) | 114.5; 120.0 (48.6) | 7.5 (–5.1, 20.1) | 100.0; 110.4 (63.5) | 114.0; 119.5 (63.8) | 12.2 (–0.5, 24.9) |
| Procedure time  Median; mean (SD) | 53.5; 64.6 (43.2) | 57.0; 61.8 (41.0) | –4.1 (–12.4, 4.2) | 55.0; 63.9 (42.2) | 55.0; 63.5 (43.4) | –0.8 (–9.2, 7.5) |
| Door to groin  Median; mean (SD) | 114.0; 127.3 (77.7) | 152; 164.6 (97.3) | 30.9 (15.5, 46.3) | 118.0; 134.2 (85.4) | 140.5; 148.4 (85.3) | 17.2 (1.6, 32.7) |
| Door to procedure end  Median; mean (SD) | 175.0; 195.3 (100.1) | 211.0; 228.3 (114.2) | 24.4 (5.0, 43.8) | 181.0; 201.2 (104.5) | 194.5; 214.3 (107.5) | 16.4 (–3.1, 35.8) |

CI = confidence interval; SD = standard deviation.

End of procedure is defined as the time of the final intracranial angiogram.

* Denominators differ between variables owing to missing or not applicable values.

** Out-of-hours minus in-hours. Adjusted for age, sex, hypertension, atrial fibrillation, hyperlipidaemia, diabetes mellitus, previous stroke/TIA, coma, NIHSS at onset, anterior/posterior circulation lesion.

*** In total 40 patients were transferred straight to the neurointervention suite and received no imaging at UHNM prior to thrombectomy.

# Table S3 Details of the procedure, day versus night and weekday versus weekend

|  | **Day vs night** | | | **Weekday vs weekend** | | |
| --- | --- | --- | --- | --- | --- | --- |
|  | **Day (n=354)** | **Night (n=147)** | **Adjusted odds ratio (95% CI)*** | **Weekday (n=359)** | **Weekend (n=142)** | **Adjusted odds ratio (95% CI)**** |
| CT perfusion; n (%) | 46 (13.0) | 10 (6.8) | 0.51 (0.24, 1.07) | 41 (11.4) | 15 (10.6) | 0.95 (0.50, 1.81) |
| General anaesthetic; n (%) | 261 (76.1) | 110 (79.7) | 1.22 (0.74, 2.02) | 269 (78.2) | 102 (74.5) | 0.71 (0.44, 1.15) |
| Successful recanalization; n (%) | 309 (71.7) | 122 (28.3) | 0.57 (0.32, 1.02) | 313 (88.4) | 118 (84.9) | 0.70 (0.39, 1.26) |
| First pass recanalization; n (%) | 156 (48.1) | 67 (49.3) | 1.00 (0.65, 1.52) | 154 (46.5) | 69 (53.5) | 1.29 (0.84, 1.96) |
| Intracranial vasospasm; n (%) | 6 (1.7) | 1 (0.7) | 0.41 (0.05, 3.59) | 3 (0.8) | 4 (2.8) | 3.27 (0.67, 15.94) |
| Dissection; n (%) | 25 (7.1) | 8 (5.5) | 0.68 (0.29, 1.60) | 24 (6.7) | 9 (6.4) | 0.89 (0.40, 1.99) |

Percentages are valid percentages (based on non-missing values) Successful recanalization was defined as a thrombolysis in cerebral infarction (TICI) score of 2b or 3.

* Day as reference category. An odds ratio greater than 1 indicates that the outcome was more likely among night patients than among day patients, and an odds ratio less than 1 that it was less likely.

** Weekday as reference category. An odds ratio greater than 1 indicates that the outcome was more likely among weekend patients than among or weekday patients, and an odds ratio less than 1 that it was less likely.

# Table S4 Complications and outcomes up to 90 days, day versus night and weekday versus weekend

|  | **Day vs night** | | | **Weekday vs weekend** | | |
| --- | --- | --- | --- | --- | --- | --- |
|  | **Day (n=354)** | **Night (n=147)** | **Adjusted difference (95% CI)*** | **Weekday (n=359)** | **Weekend (n=142)** | **Adjusted difference (95% CI)*** |
| NIHSS at 1 week; median; mean (SD) (IQR) | 6.0; 12.4 (13.6) | 8.0; 14.5 (14.7) | 1.5 (–1.1, 4.0) | 7.0; 12.6 (13.6) | 8.5; 14.2 (14.8) | 1.2 (–1.3, 3.8) |
| Renal failure; n (%) | 8 (2.3) | 0 (0.0) | ** | 5 (1.4) | 3 (2.1) | 2.49 (0.46, 13.45) |
| Groin haematoma; n (%) | 7 (2.0) | 1 (0.7) | 0.41 (0.05, 3.56) | 6 (1.7) | 2 (1.4) | 0.83 (0.16, 4.31) |
| Malignant middle cerebral artery syndrome; n (%) | 30 (8.5) | 14 (9.5) | 1.14 (0.53, 2.45) | 23 (6.4) | 21 (14.9) | 2.31 (1.15, 4.63) |
| Hemicraniectomy; n (%) | 9 (2.5) | 4 (2.7) | 1.13 (0.28, 4.50) | 8 (2.2) | 5 (3.5) | 1.22 (0.32, 4.64) |
| Symptomatic intracerebral haemorrhage; n (%) | 6 (1.7) | 2 (1.4) | 0.81 (0.15, 4.27) | 5 (1.4) | 3 (2.1) | 1.49 (0.34, 6.53) |
| Subarachnoid haemorrhage; n (%) | 31 (9.2) | 14 (9.8) | 1.10 (0.55, 2.19) | 33 (9.6) | 12 (8.8) | 0.92 (0.45, 1.86) |
| Deep vein thrombosis within 90 days; n (%) | 9 (2.6) | 3 (2.0) | 0.92 (0.23, 3.69) | 9 (2.5) | 3 (2.1) | 0.83 (0.22, 3.24) |
| Pulmonary embolism within 90 days; n (%) | 13 (3.7) | 2 (1.4) | 0.33 (0.07, 1.55) | 10 (2.8) | 5 (3.5) | 1.31 (0.42, 4.07) |
| Stroke within 90 days; n (%) | 6 (1.7) | 4 (2.8) | 1.75 (0.46, 6.61) | 5 (1.4) | 5 (3.6) | 2.59 (0.71, 9.41) |
| Death at 90 days; n (%) | 63 (17.8) | 33 (22.4) | 1.42 (0.83, 2.42) | 63 (17.5) | 33 (23.2) | 1.59 (0.94, 2.69) |
| Functional independence at 90 days; n (%) | 170 (48.0) | 64 (27.4) | 0.82 (0.53, 1.25) | 175 (48.7) | 59 (41.5) | 0.74 (0.49, 1.14) |

Percentages are valid percentages (based on non-missing values)

Functional independence is defined as s modified Rankin Scale score of 0-2. Symptomatic intracerebral haemorrhage is defined as per the SITS-MOST criteria.

* Difference expressed as odds ratio (with reference categories day for day versus night and weekday for weekday versus weekend), except for NIHSS, where it is mean difference (day minus night and weekend minus weekday)

** Odds ratio not calculable
